# The Use of Artificial Intelligence Chatbots by Newly Diagnosed Cancer Patients: A Descriptive Phenomenological Study

**DOI:** 10.3390/curroncol33090563

**Published:** 2026-09-17

**Authors:** Mustafa Serkan Alemdar, Yağmur Çolak, İlhan Günbayı, Hasan Mutlu

**Affiliations:** 1 Department of Medical Oncology, Istinye University, Istanbul 34396, Türkiye; 2 Department of Medical Oncology, Medical Park Hospital, Antalya 07160, Türkiye; 3 Department of Psychiatric Nursing, Faculty of Nursing, Akdeniz University, Antalya 07070, Türkiye; 4 Department of Educational Administration, Faculty of Education, Akdeniz University, Antalya 07070, Türkiye

**Keywords:** cancer diagnosis, artificial intelligence, chatbot, patient experience, qualitative research

## Abstract

After a cancer diagnosis, patients experience intense uncertainty and increasingly resort to artificial intelligence chatbots in this process; however, how this use is experienced by patients has not been adequately studied. This study aimed to explore the experiences of adult patients diagnosed with cancer in the last six months using artificial intelligence (AI) chatbots. Using a descriptive phenomenological approach, face-to-face semi-structured interviews were carried out with 20 adults sampled by a criterion-based purposive sampling method in an oncology clinic in Türkiye. The data were analyzed according to Colaizzi’s method, and the following four main themes were revealed: artificial intelligence usage purposes, the experience of interacting with AI, the effect of the patient’s reflection on the AI experience on the relationship with the treatment team, and evaluation of the use of AI. The participants used chatbots as an intermediate resource during the period of uncertainty between the examination result and clinical explanation; some learned their diagnosis for the first time in this way. However, the relief provided by chatbots was temporary, and trust in chatbot results was constantly tested by the statements of the healthcare professional. As a result, some participants hid their use of chatbots for fear of being judged. AI chatbots served as a complementary source of information, but they did not replace the relationship with the healthcare professional. These findings suggest that healthcare professionals should ask patients about chatbot use without judgment, and that support provided during the post-examination waiting process could be strengthened.

## Simple Summary

A cancer diagnosis is an experience that overwhelms an individual with uncertainty and anxiety, and the process of obtaining a clinical explanation is often navigated in isolation. In recent years, an increasing number of patients have been using artificial intelligence (AI) chatbots to cope with this uncertainty. In this study, 20 adults diagnosed with cancer in the past six months were interviewed to analyze why and how they use AI tools. Patients used chatbots to understand medical terms, find quick answers to their questions about the treatment process, and prepare for a physician’s consultation, and some learnt their diagnosis for the first time. One key finding is that the relief provided by using AI was temporary, and lasting reassurance came mainly from the physician’s explanation. The participants did not trust artificial intelligence unconditionally, comparing the answers they received with those from their physicians, and some suppressed this use for fear of being judged. These findings suggest that healthcare professionals should consider asking patients about chatbot use without judgment, and that support provided during the post-examination waiting process could be strengthened. This approach can contribute to patients feeling more secure and supported during times of uncertainty.

## 1. Introduction

A cancer diagnosis can lead to significant physical, psychological, and social changes in individuals’ lives. During the diagnosis and treatment process, patients require comprehensive information and psychosocial support in understanding the disease and treatment options, obtaining information about the possible course of the disease, communicating effectively with healthcare professionals, and coping with the emotional effects of the disease [1]. In line with these requirements, many patients refer to online resources in addition to the information presented in clinical interviews. By providing quick access to information, these resources can support disease understanding and patient involvement in care decisions. However, the level of utilization of online resources depends on factors such as digital literacy, the perceived reliability of the information, and the quality of the relationship established with the healthcare professional [2]. Additionally, the process of searching for information online may result in patients encountering inaccurate, contradictory, or alarming content. Repetitive, reassurance-seeking information behavior has also been associated, though not causally, with health anxiety and cyberchondria [3].

In this context, large language model (LLM)-based chatbots stand out as an interactive form of online health information search. In a web-based study, 21.2% of respondents reported using an LLM-based chatbot to gain health information in the past year. The use of LLM-based chatbots has been found to be more common in younger individuals and those who are positive about AI [4]. While these tools are easily accessible and interactive, in addition to their ability to generate contextually appropriate responses to questions, they introduce elements related to perceived benefit, reliability, and risk [5]. Because of these features, conversational artificial intelligence (AI) offers a different interaction than a traditional internet search and requires a separate examination of its role in patients’ information-seeking experiences.

AI chatbots in oncology have been examined as complementary tools for delivering cancer information, strengthening health literacy and self-management, and offering emotional support [6]; moreover, in some comparative studies, the written responses of chatbots have been rated as more empathetic than those of physicians [7,8]. However, these potential benefits are accompanied by significant limitations. Since these findings are based on written chatbot responses isolated from context, chatbots should not be viewed as a substitute for human empathy or a replacement for relational communication with a healthcare professional. A meta-analysis involving 56 oncology studies reported an average overall chatbot response accuracy of 76.2% and a diagnostic accuracy of 67.4%, revealing infrequent evaluation of safety and vulnerability dimensions [9]. In a study on lung cancer radiotherapy, although clinicians and patients found chatbot responses helpful, objective readability analyses found the texts mostly difficult to read [10]. Broader concerns include misleading outputs, privacy, absence of human empathy, and the risk of depersonalizing care [8,11]. Therefore, it is necessary to understand how patients position themselves between the benefits and concerns related to the use of chatbots.

Although research on AI chatbots in cancer care is growing rapidly, much of the literature has evaluated the accuracy, quality, and readability of responses rather than patients’ experiences using these tools in everyday life. A small number of qualitative studies examined the experience of cancer survivors using existing tools [12], reactions to hypothetical scenarios [13], or the perception of ChatGPT as a general source of health information [14]; as a whole, these studies focus on acceptability, trust, and usability. However, the use of chatbots takes place within the context of a patient’s clinical relationship, thus bringing with it the question of whether the chatbot use is shared with the treatment team. Therefore, relatively little is known about how patients spontaneously incorporate these tools into their lives after diagnosis, what triggers their use, how they evaluate the responses they receive, and what meanings they attribute to these interactions, sometimes in metaphorical terms. Because communication and information needs in cancer care vary across the stages of the care journey [15], the first post-diagnosis period is of particular importance: patients face intense uncertainty regarding diagnosis, stage, and treatment while awaiting clarification from the clinical team, which may differentiate their experience from that of survivors studied to date [12].

Accordingly, this study aimed to examine the experiences of patients diagnosed with cancer in the last six months using artificial intelligence chatbots in depth with a descriptive phenomenological approach. In this context, the main research question is as follows:

- “What is the essence and meaning of the experiences of adult patients diagnosed with cancer regarding the use of AI chatbots?”

## 2. Method

### 2.1. Research Design

This study was carried out with a descriptive phenomenological approach to examine the experiences of patients diagnosed with cancer in the last six months using artificial intelligence chatbots in depth. Descriptive phenomenology aims to describe experiences related to a phenomenon based on the narratives of the participants and to reveal the common essences of their experiences [16,17]. In the analysis of the data, Colaizzi’s descriptive phenomenological analysis method was followed [18]. The reporting of this study was based on the Consolidated Criteria for Reporting of Qualitative Research (COREQ) checklist [19].

### 2.2. Research Setting and Participants

This study was carried out in an oncology clinic. Patients who presented to the clinic for follow-up or treatment during the recruitment period and who met the age and diagnosis criteria were identified by the research team; the interviewer (M.S.A.) informed each patient about this study and asked verbally whether they had used an artificial intelligence chatbot at least once since their cancer diagnosis. The participants were selected using a criterion-based purposive sampling method. The criteria for inclusion in the study were as follows: age of 18 years or older, a histopathologically confirmed cancer diagnosis in the last six months, use of an artificial intelligence chatbot at least once after cancer diagnosis, able to communicate in Turkish, and voluntary agreement to participate in the study. Having a cognitive or communicative problem that would prevent the patient from participating in the interview was an exclusion criterion. Seven of the 27 patients evaluated regarding the inclusion criteria were not included in this study because they had not used an artificial intelligence chatbot after cancer diagnosis; therefore, data collection was completed with 20 participants.

### 2.3. Ethical Considerations

This study was approved by the Human Research Ethics Committee of Istinye University (meeting date: 15 May 2026; protocol no.: 2026/149). Institutional permission was obtained from Antalya Medical Park Hospital. The participants were given verbal and written information about the purpose of the research, audio recording, confidentiality principles, and their right to withdraw from this study, and their written informed consent was obtained. It was communicated that the decision to participate in this study would not affect the treatment and care processes of the patients. The participants were anonymized with K01–K20 codes, and audio recordings and transcripts were stored in secure digital media that only the research team had access to. This study was conducted in accordance with the Declaration of Helsinki [20].

### 2.4. Data Collection

Data were collected through semi-structured individual interviews in July 2026. The interviews were carried out by an oncology specialist trained in qualitative interview techniques in the oncology clinic. The interview guide consisted of open-ended questions addressing the purposes of using artificial intelligence, interaction and emotional experiences, trust, reflections on relationships with healthcare professionals, general evaluation of use, and metaphorical expressions. The interviews with the twenty participants lasted an average of 25.7 min (SD = 3.3) each and were recorded with the permission of the participants. During the process of conducting the interviews, the fatigue and treatment burden experienced by participants receiving oncological treatment were taken into consideration. The audio recordings were transcribed verbatim, and the transcriptions were anonymized. The transcription and analysis processes were carried out by a psychiatric nurse and an academician who is an expert in qualitative research. The accuracy of the transcriptions was verified by comparing the written texts with the audio recordings. Data collection and preliminary analysis were carried out simultaneously. After the first 16 interviews, it was observed that no new code, meaning pattern, or sub-theme emerged; therefore, four additional interviews were conducted to evaluate the adequacy of the existing analytical structure. Since these interviews did not add a new dimension to the existing structure, it was decided that data saturation was reached [21,22]. Code and meaning saturations were considered in reaching this decision: code saturation, in the sense of Guest et al. [21], was indicated by the absence of new codes or sub-themes in the final interviews, whereas meaning saturation, in the sense of Hennink et al. [22], was indicated by the absence of new nuances, dimensions, or depth within the already-identified sub-themes; that is, the four additional interviews did not enrich or complicate the understanding of the existing analytical structure. Both forms of saturation were judged to have been reached concurrently at 20 interviews.

### 2.5. Data Analysis

The data were analyzed according to Colaizzi’s (1978) seven-step protocol [18]. In this direction, the verbatim transcripts were read holistically, meaningful expressions related to the investigated phenomenon were determined, formulated meanings were created, similar meanings were combined under theme clusters, a comprehensive description was written, and the basic structure of the experience was developed. This final step was carried out as an interpretive synthesis rather than a further categorization implementation: the coding researchers integrated the four main themes into a single exhaustive description of participants’ experience and distilled from it a statement of the fundamental structure of the phenomenon, which is presented at the end of Section 3 and revisited in Section 4, to convey the lived experience as a whole rather than only its constituent codes. The anonymized verbatim transcripts were imported into the licensed MAXQDA 26.02 software of the university to which the two researchers were affiliated. A psychiatric nurse and an academician who is an expert in qualitative research read and coded the transcripts independently of each other. After independent coding, the researchers compared the codes and the formulated meanings, and the differences were discussed and agreed upon by returning to the raw data. When the two coders’ initial codes or formulated meanings diverged, they met to discuss each discrepancy jointly and re-examined the corresponding segment of the verbatim transcript in its original context. Disagreements were resolved through this negotiated, evidence-based discussion in every instance, and no case required arbitration by a third team member. As a result, the final analytical structure consisting of four main themes and 10 sub-themes/codes was obtained, and the total dataset was re-checked with the final codebook. To evaluate the analytical structure created from the outside, the opinion of a third academician who was not involved in the analysis process and is an expert in the field of qualitative research was taken. For this purpose, an evaluation table consisting of 10 anonymous statements was prepared by selecting one participant statement representing each sub-theme among the codes agreed upon by the two coders. The expert was presented with this table and the codebook containing the definitions of the sub-themes, and was then asked to assign each statement independently to one of the 10 sub-themes. Full agreement was found between the assignments of the expert and the reference coding of the researchers (κ = 1.00). This procedure was designed as a targeted external auditor check on the clarity and discriminant validity of the codebook; that is, the procedure determined whether an independent expert who had not taken part in coding could correctly allocate one example quotation per sub-theme using only the sub-theme definitions, rather than as a comprehensive measure of inter-rater reliability across the full dataset. The primary safeguard for coding consistency across all 20 transcripts remained the independent double-coding and consensus-based resolution of disagreements described above.

Within the scope of the seventh phase of Colaizzi, all 20 participants were contacted by phone and invited for participant confirmation. Face-to-face confirmation interviews were conducted with 12 participants (60%) who were able to spare time during routine follow-up or treatment visits at the hospital. The participants were presented with the basic structure of the research and a summary of the four main themes and 10 sub-themes prepared in everyday language. The participants were asked whether the findings reflected their own experiences and whether there was anything that was missing or incorrect. All 12 participants stated in the confirmation interview that the findings were consistent with their experiences, and no new theme or sub-theme emerged as a result of the feedback.

### 2.6. Rigor

The scientific rigor of the research was ensured in line with the criteria of Lincoln and Guba for credibility, transferability, dependability, and confirmability [23]. The credibility of this study was supported by the participation of two researchers in the analysis, expert evaluation, and participant confirmation. The research environment and participant characteristics were described in detail for transferability, and the findings were supported by direct quotes from different participants and through in-depth description and detailed contextualization of participant backgrounds, experiences, and study contexts. Dependability was achieved through the systematic analysis process, the use of the codebook, and the recording of analysis decisions. For confirmability, the chain of analysis from raw data to codes, sub-themes, and themes was documented, and reflective notes were kept. These reflective notes also served a bracketing (epoché) function: throughout coding and analysis, the researchers who coded the data recorded and consciously set aside their own prior assumptions about artificial intelligence and physician authority so that the analytic categories would emerge from participants’ narratives rather than from the researchers’ preconceptions. During the analysis, it was taken into account that the interviewer’s clinical care relationship with some participants could affect the expressions, potentially introducing an authority effect or social desirability bias, for example, a reluctance to criticize physicians or disclose chatbot use. This possible effect was minimized by explicitly emphasizing voluntariness, using a non-judgmental interviewing style that explicitly invited participants to share critical views of their physicians or their chatbot use without fear of judgment, using standard interview guidelines, and having the interview transcription and analysis conducted by investigators who were not involved in the clinical care of the participants. This separation between interviewing and coding was itself designed as a bracketing strategy to prevent the interviewer’s perspective from shaping the analytical process.

## 3. Results

In this section, the sociodemographic, clinical, and technology usage characteristics of the participants are presented. The themes and sub-themes obtained from the analysis of the interviews are also included; the analysis was carried out with a descriptive phenomenological approach to examine the experiences of using artificial intelligence chatbots and the metaphorical expressions produced by the participants.

When the characteristics of the participants were examined, the age range was 34–79 and the average age was 52.4. A total of 12 (60%) of the participants were female and eight (40%) were male. The most common diagnosis was breast cancer (*n* = 12, 60%), and 14 (70%) of the participants had stage I or II disease (Table 1).

A total of 90% of respondents reported using digital devices on a daily basis, and ChatGPT was the most commonly used AI tool (70%). Although 70% of the participants started using artificial intelligence before the diagnosis of cancer, 60% reported that its use increased after the diagnosis (Table 2).

The experiences of patients interacting with AI chatbots in the cancer care process are centered around the following four main themes: (1) artificial intelligence usage purposes, (2) the experience of interacting with AI, (3) the effect of the patient’s reflection on the AI experience on the relationship with the treatment team, and (4) the evaluation of the use of artificial intelligence (AI). Theme 1 explains patients’ use of artificial intelligence as their first source of information acquisition and their purpose in using these tools in the treatment process and in the management of side effects. Theme 2 addresses the emotional effects of AI use on patients and their level of trust when interacting with AI. Theme 3 explains how the artificial intelligence experience is reflected in a patient’s relationship with healthcare professionals through the vitalness of the healthcare professional, the patient’s preparedness upon attending the interview, and the dynamic of sharing the AI usage experience. Theme 4 covers the perceived benefits of the use of AI, the limitations of the use of AI, and the AI usage recommendations in the same process. All four main themes were present in the narratives of every participant; the participant-level distribution of sub-themes is provided in Supplementary Table S1. The findings are presented below theme by theme, supported by participant statements.

**Theme 1: Artificial intelligence usage purposes**

The participants’ purpose of using artificial intelligence chatbots was shaped around two basic patterns: (1) using artificial intelligence as the first source of information acquisition and (2) using artificial intelligence in the management of the treatment process and side effects. The participants used artificial intelligence as a complementary resource that made sense of information immediately after they received the results of their examination, reduced uncertainty until they could consult with their healthcare professional, and provided quick answers to daily questions that arise during treatment.

**Sub-theme 1: Artificial intelligence as the first source of information acquisition**

Participants turned to artificial intelligence to explain the medical terms in reports on pathology, biopsy, PET, MRI, ultrasound, and blood test results, and to learn what the findings mean. This use mostly took place in the time interval between learning the test result and its announcement by the healthcare professional.

A participant who uploaded the biopsy report to artificial intelligence stated that she learned her diagnosis for the first time in this way:

*“Of course, I have already learned the diagnosis from artificial intelligence. Because I didn’t know the results of the reports and what the medical terms meant, I uploaded my first biopsy report to artificial intelligence and asked it to interpret it for me. And I learned firsthand that I had cancer directly from there. Then I made my physician’s appointment.”*

(K17; 42 years, female; breast cancer, stage I)

Some participants used chatbots to learn the possible causes of the symptoms they noticed and determined the first step to take before the clinical evaluation was conducted.

*“After realizing that I had any mass on my chest, the first thing that came to my mind was Chat GPT because I noticed it the evening. I asked it to come up with positive and negative versions of what could happen and to ask questions about which surgeon or department I should go to as the first intervention I should do.”*

(K03; 47 years, female; breast cancer, stage II)

These narratives demonstrate that chatbots are experienced as an initial resource that enables patients to access information at the first moment of uncertainty and structure the subsequent healthcare application. The information obtained from artificial intelligence was considered temporary and complementary information that was mostly sought before the interview with the healthcare professional.

**Sub-theme 2: Treatment process and management of side effects**

The focus of the questions posed to artificial intelligence after diagnosis ranged from understanding what the disease was and how treatment would proceed to evaluating whether the symptoms experienced were an expected side effect. The participants used chatbots to learn about chemotherapy and surgery processes, the effects of drugs, blood values, nutrition, and preparation for tests, as well as pain, diarrhea, and other daily symptoms.

In particular, participants turned to AI for minor symptoms that they considered not urgent or did not require repeated consultation with a healthcare professional.

*“It’s just the things that I need, the side effects of the treatments that I’ve taken little by little, so are these side effects normal? For example, I have a headache, is this a normal pain? I have diarrhea, is this a side effect of normal chemotherapy? You know, instead of constantly disturbing my physician, I researched some small things like this through artificial intelligence.”*

(K05; 41 years, female; breast cancer, stage II)

The function of artificial intelligence in this process became more evident in situations in which healthcare professionals could not be reached immediately. The participants stated that the AI answers they received helped them understand the possible causes of their symptoms and established the relationship between their condition and treatment. A participant who asked artificial intelligence about the pain she experienced during chemotherapy shared this experience as follows:

*“I was relieved by the answers it gave to me, especially in questions about side effects, such as my pain during chemotherapy. So I understood what the reason was. That’s why I calmed down. When I couldn’t reach the physician right away, I used artificial intelligence. And the answers I received in that process relieved me.”*

(K13; 43 years, female; breast cancer, stage II)

The accounts provided by the participants suggest that the purpose of using artificial intelligence varied according to different stages of the disease process. In the first stage, the questions “What does this result mean?” and “Which health unit should I consult?” came to the fore, while with the start of treatment, the questions turned to “What awaits me?” and “Is this symptom normal?”

**Theme 2: The experience of interacting with AI**

Participants’ interactions with chatbots were shaped around two key patterns: (1) the emotional effects of using AI and (2) trust in AI interactions. The accounts provided by the participants suggest that AI interaction was not entirely relaxing or anxiety-inducing, and that the emotional impact varied depending on the content of the response, the way it was presented, the time of use, and the explanation received from the healthcare professional afterwards. Similarly, trust emerged as a conditional assessment that developed as a result of comparing the responses received with the statements of the healthcare professional.

**Sub-theme 3: Emotional effects of using artificial intelligence**

Participants stated that the responses they received from artificial intelligence sometimes provided relief by reducing uncertainty, and sometimes increased fear and anxiety by presenting negative possibilities in a context-independent manner. While learning the possible cause of symptoms, encountering a positive detail in the test report or obtaining an explanation until reaching a healthcare professional provided temporary relief. Encountering the word cancer directly on the screen or viewing a list of the most negative possibilities and responses without taking into account the personal clinical situation could result in crying, insomnia, and intense anxiety.

The fact that artificial intelligence responses present heavy possibilities without a holistic evaluation led some participants to abandon health-related research altogether. A participant described the anxiety she experienced after the response she received from artificial intelligence and the change in her usage behavior after the healthcare professional’s explanation as follows:

*“It’s too bad, I couldn’t go to bed until the morning. But when I went to my physician, he or she said more beautiful things that comforted me. I never ask ChatGPT or Google about a disease anymore.”*

(K10; 42 years, female; breast cancer, stage I)

On the other hand, some participants stated that the preliminary information they received from artificial intelligence provided temporary relief during the period when they were waiting for the examination result to be announced.

*“For example, my report falls into the system, for example, I take a screenshot from there and have it interpreted from ChatGPT. Some time must have passed until the appointment. At that time, I have already interpreted it. Frankly, there is a preliminary relief. But hearing it from my physician’s mouth is a completely different feeling.”*

(K12; 34 years, female; breast cancer, stage II)

These narratives demonstrated that the emotional relief provided by AI was often “pre-relief.” AI was able to reduce the uncertainty of the waiting time, but it was mostly after a consultation with a healthcare professional that participants were permanently relieved and emotionally certain about their clinical condition.

**Sub-theme 4: Trust in AI interaction**

Instead of accepting the information received from artificial intelligence as directly correct, the participants compared it with the statements of the healthcare professional, scientific sources, or different sources of information. While the fact that the AI answers coincided with the opinions of the healthcare professional increased trust in artificial intelligence, trust was limited by the fact that different answers were received in response to the same question, the sources used were not known, and the answer depended on the way the question was expressed.

One of the important conditions of trust for the participants was that the problem was established correctly and with sufficient detail. Stating that an incorrect or incomplete question could produce confusing results, one participant indicated that artificial intelligence knowledge should be evaluated together with a healthcare professional:

*“First of all, it is a system that always confuses me when the right question is not asked to the artificial intelligence about my disease. I am absolutely determined that the most accurate data is to share the information you receive from the artificial intelligence that carries out that treatment protocol with your physicians and find the right way. So that point is very important. Artificial intelligence is right, there is no such thing as yes.”*

(K07; 66 years, male; sarcoma, stage I)

The recurring alignment between AI responses and healthcare professional explanations led some participants to trust AI more over time.

*“I was first looking unconsciously. I had no idea if it was true or not. But for example, after meeting with my physician a few times and generally getting the same results, my confidence in artificial intelligence increased. Because it was usually saying what my physician said. That’s how I started consulting more AI. But I still found what my physician said more important. I trusted him more, and I still trust him more.”*

(K12; 34 years, female; breast cancer, stage II)

The accounts provided by the participants suggest that trust in AI was more of a variable relationship established by continuous validation and comparison than one of unlimited acceptance. Participants’ trust in AI increased when responses resonated with previous experiences and the healthcare professional’s explanations; conversely, trust in AI decreased when participants were faced with contradictory, generalizing, or unsourced responses.

**Theme 3: The effect of the patient’s reflection on the artificial intelligence experience on the relationship with the treatment team**

The theme of the effect of the patient’s reflection on the artificial intelligence experience on the relationship with the treatment team consisted of three basic patterns: (1) the vitalness of the healthcare professional, (2) the patient’s preparedness upon attending the interview, and (3) the dynamic of sharing the AI usage experience. Although the participants benefited from artificial intelligence, it positioned the healthcare professional at the center of the treatment process regarding the clinical decision, physical evaluation, personalized explanation, and emotional communication. While AI enabled some participants to prepare more consciously for the interview, sharing AI usage with the treatment team varied based on the expected or experienced response from the healthcare professional.

**Sub-theme 5: The vitalness of the healthcare professional**

The participants defined a healthcare professional not only as a person who verified the information received from artificial intelligence but also as a person who performed a physical examination, evaluated the patient’s clinical history and responded to treatment, considered physiological and psychological characteristics together, and assumed clinical responsibility. The main differences were that artificial intelligence can only respond to the information submitted to it; AI cannot observe the patient or bear the responsibility of the clinical decision.

One participant evaluated the healthcare professional’s recognition of the patient regarding their biological, physiological, and psychological aspects as the main feature that distinguished the healthcare professional from artificial intelligence:

*“When I talk to my physician, my physician knows me from start to finish, he or she knows my physiological structure, he or she knows my biological structure, he or she knows my psychology very well. Because even though I hide something, as a physician, I think he understands very well what I am suppressing from my facial expression while I am telling it. But we cannot provide this in artificial intelligence. Whatever I write about artificial intelligence, it gives me the answer. But whatever I ask the physician, the physician answers me according to what he sees in front of him.”*

(K05; 41 years, female; breast cancer, stage II)

The superiority of the healthcare professional was explained not only through clinical knowledge and personalization but also through the ability to present negative information in a more emotionally sensitive manner. One participant noted that even if the same information was provided by AI and a healthcare professional, the way the information was conveyed created a different experience:

*“Because a machine will answer me, they have no feelings. Since it has no feelings, its clear answer may upset me. But I believe that my physician will be able to motivate me a little more by getting me used to it a little more. I know that the physician will soften me a little and prepare me until he or she gives that answer. Even if the answer is bad. Maybe the answers given are the same. But the machine tells me that directly.”*

(K16; 49 years, male; gastric cancer, stage III)

The accounts provided by the participants suggest that the speed and information access provided by AI were not seen as equivalent to personalized clinical assessment, accountability, and human contact. The use of artificial intelligence contributed to making the clinical and relational value of the healthcare professional more visible.

**Sub-theme 6: The patient’s preparedness upon attending the interview**

The prior knowledge gained from AI helped patients to ask more focused questions in the limited interview time and to discuss issues related to the personal clinical situation rather than repeating basic information.

One participant stated that the use of artificial intelligence changed the nature of the questions she asked the healthcare professional:

*“I am going more consciously about myself, about my illness. I am going by learning something. I ask the physician more carefully the question I will ask, and I go accordingly more consciously.”*

(K13; 43 years, female; breast cancer, stage II)

The accounts provided by the participants suggest that AI was experienced by some participants as a complementary tool that provided cognitive preparation for the health interview, helped filter questions, and supported more effective patient participation in the interview.

**Sub-theme 7: The dynamic of sharing the AI usage experience**

Some participants openly shared with healthcare professionals the fact that they used artificial intelligence or the information they obtained from it and encountered positive or neutral reactions. Others, out of concern for being rejected, belittled, or appearing to disrespect the expertise of the healthcare professional, did not mention its use, but carried the information they learned from artificial intelligence to the interview without specifying its source.

One participant, who openly shared the information he received from artificial intelligence, described the initial rejection reaction of the healthcare professional:

*“After that, the physician’s brain reaction threw the paper in front of me. Then he said chatGPT should check you. He reacted like this.”*

(K02; 55 years, male; gastric cancer, stage I)

On the other hand, some respondents stated that healthcare professionals considered the use of artificial intelligence as a current and normal behavior and that they also benefited from these tools when necessary:

*“He responded positively about it and even told us that he used it when necessary.”*

(K01; 62 years, male; breast cancer, stage II)

While an accepting or neutral approach made it easier to evaluate the information obtained from artificial intelligence together, the expectation of judgment or rejection led some patients to conceal the use of AI from the healthcare professional.

**Theme 4: Evaluation of the use of artificial intelligence (AI)**

The evaluation of the use of artificial intelligence consisted of three sub-themes: (1) the perceived benefits of AI, (2) the perceived limitations of AI, and (3) the AI usage recommendations in the same process. While the previous themes described the participants’ experience with artificial intelligence, this theme included the retrospective evaluation of the same experience by the participants and the recommendations derived from this evaluation. In general, the participants evaluated artificial intelligence within the framework of a conditional benefit. While quick access and clarity of medical information stand out as significant advantages, the lack of personalization, reliability issues, and the absence of human context were cited as key limitations.

**Sub-theme 8: Perceived benefits**

The most obvious benefit of the use of AI was the ability to receive quick responses at any time of the day, providing patients with access to information without having to wait to reach a healthcare professional. The participants also found that they could ask the same question again, collect multiple types of information in a short time, and re-examine the answers. Simplification of medical terms, understanding of reports, and referral to the appropriate health unit were also among the important contributions.

One participant expressed this situation as follows:

*“The most useful aspect is quick, quick answer… If the question is clear, the answer is clear. That’s why that’s the benefit of artificial intelligence: instant answers.”*

(K16; 49 years, male; gastric cancer, stage III)

Another participant noted the ease AI provides in understanding medical reports:

*“I couldn’t decipher foreign terms in pathology and blood tests… When the artificial intelligence explained it, I was able to understand what it was.”*

(K09; 67 years, female; breast cancer, stage I)

Overall, the findings suggest that AI is considered a tool that accelerates access to information and prepares patients for a health interview.

**Sub-theme 9: Perceived limitations of AI**

The limitations reported in this sub-theme reflect participants’ own perceptions and subjective experiences of AI chatbot responses, rather than an independent, objective evaluation of chatbot accuracy conducted by the research team. The most important limitations are the lack of personalization of responses, not taking into account clinical history, and the inability to perform physical examination. The limitations also highlighted the risk of AI chatbots generating different and contradictory responses, source uncertainty, and misinformation.

One participant explained this situation as follows:

*“There is information overload on the internet, and artificial intelligence can give it without filtering. So it can’t be trusted directly.”*

(K17; 42 years, female; breast cancer, stage I)

Another participant noted the lack of a human dimension:

*“A physician can see and understand what I feel… Artificial intelligence does not have this.”*

(K18; 58 years, female; breast cancer, stage III)

The accounts provided by the participants suggest that the main perceived limitation was misinformation as well as the presentation of information that was disconnected from clinical and emotional contexts; this study did not independently verify the factual accuracy of the AI outputs described by participants.

**Sub-theme 10: AI usage recommendations in the same process**

The dominant view on AI usage recommendations was that AI could be used for information acquisition, term clarification, and interview preparation; however, it must be verified by a healthcare professional. Correct questioning, source comparison, and critical evaluation were stated as important terms of use.

One participant summarized this approach as follows:

*“It is useful for initial information, but it must be confirmed with an expert.”*

(K01; 62 years, male; breast cancer, stage II)

In contrast, some respondents suggested consulting a healthcare professional directly instead of AI, especially due to anxiety-inducing experiences:

*“They should not do research for the disease; they should go directly to the physician.”*

(K10; 42 years, female; breast cancer, stage I)

**Fundamental structure of the experience**

When the themes are evaluated together, the use of artificial intelligence during and after the cancer diagnosis process has emerged as an experience of interaction with a complementary intermediate source that provides fast and understandable information in times of uncertainty. However, the reliability of AI needs to be verified by a healthcare professional, and it does not replace the clinical relationship. In its fundamental structure, the experience of using an AI chatbot after a cancer diagnosis can be described as follows: patients turn to the chatbot at moments of acute informational uncertainty, most often in the interval between receiving a test result and its clinical explanation, seeking a rapid and comprehensible, if provisional, answer. The relief the use of an AI chatbot provides patients is temporary and remains contingent on subsequent confirmation by a healthcare professional, whose personalized, embodied, and accountable presence patients continue to regard as irreplaceable. This dependence on the human relationship coexists, often uneasily, with a reluctance to disclose chatbot use for fear of being judged. Therefore, AI technology could be woven into the illness experience as a provisional, closely monitored companion rather than an autonomous source of authority.

**Metaphors for the use of artificial intelligence**

The meanings attributed to artificial intelligence by the participants were examined through the answers given to the question “What do you think artificial intelligence is? Is it a thing, a living thing, etc.?” in the interview form. While 19 of the participants produced a clear metaphor when describing artificial intelligence, one participant (K08) did not produce a metaphor.

Artificial intelligence has been described using various images, such as encyclopedias, fruit-bearing trees, robots, machines, powerful engines, clouds of mist, and emergency exit doors alongside human figures such as friends, therapists, doctors, teachers, and the “all-knowing aunt.” On the one hand, the participants characterized artificial intelligence through human-like figures that can be consulted, offer instruction, and provide a substitute for a sense of closeness; on the other hand, the participants defined AI through images that are emotionless, distant, of uncertain reliability, or to be turned to only in cases of necessity. The variety of metaphors showed that artificial intelligence was not perceived equivalently by the participants.

Some of the sample expressions for metaphors are as follows:

*“You know, break the window urgently, somehow… I can also say that it is an emergency exit door that can relieve us in some way, provided that I do not trust it one hundred percent absolutely.”*

(K16; 49 years, male; gastric cancer, stage III)

*“For me, it is a robot imitating a human… It is a tool that conveys the uploaded information to you.”*

(K14; 57 years, male; lung cancer, stage III)

## 4. Discussion

This study examined the experiences of patients diagnosed with cancer in the last six months using artificial intelligence chatbots with a descriptive phenomenological approach. The findings indicated that these tools were used as an intermediate source, bridging the knowledge gap between uncertainty emergence and clinical explanation. Previous qualitative studies have focused on hypothetical scenarios presented to participants without a cancer diagnosis [13], researcher-guided use [6,14], or the general experiences of cancer survivors without a requirement for prior use [12]. This study, on the other hand, dealt with the use initiated by the participants in the early post-diagnosis period and directly related to the disease in its real context.

The most prominent function of artificial intelligence after diagnosis is that it is a source of information that is used in the period between the delivery of the test result to the patient and its announcement by a healthcare professional. In a longitudinal study, it was reported that approximately half of cancer patients sought information online, and being in an early care phase was associated with this behavior [24]. Our findings suggest that early concentration may be related to the fact that the results of the examination reach the patient before clinical explanation. The participants filled this gap by having artificial intelligence interpret the screenshots of their reports made available in the hospital information system. The identification of the inadequate transfer of information as a key category in the information-seeking experiences of cancer patients [25] suggests that this behavior may also be associated with deficiencies in institutional information processes.

One of the most notable findings in this study is that some participants learned about their cancer diagnosis for the first time from the chatbot’s response after uploading their biopsy reports to AI. This suggests that chatbots are not only tools for explaining existing information but, in some cases, may become the first channel through which bad news reaches the patient. This is a striking observation from a small qualitative sample and is presented as an illustrative pattern within our specific study context rather than a generalizable claim about how cancer diagnoses are typically delivered. Clinical communication guidelines are based on the presentation of diagnostic information by a healthcare professional in an appropriate communication context [26]. Learning of diagnosis outside this context raises important questions regarding the accurate interpretation of information, the provision of emotional support, and the responsibility for communication.

The emotional impact of knowledge acquired prior to clinical explanation is twofold. While AI provided some participants with temporary “preliminary relief” that lasted until they met with a healthcare professional, responses that were out of context and focused on negative possibilities led to intense anxiety. These contrasting effects may explain the lack of a significant association between online information seeking and state anxiety [24]. Although it is known that online information seeking can become repetitive and may be associated with cyberchondria [3], this study also identified a pattern in which information-seeking behavior was completely abandoned after receiving the explanation from the physician after an alarming response. This finding suggests that clinical explanation may influence the emotional course of AI use.

Participants did not equate the empathy they perceived in chatbot responses with the empathy they experienced in the physician relationship. While there are studies reporting that chatbots are evaluated more empathetically, the findings are not consistent [7,27]. In a study in the field of cancer, the medical evaluation panel found the chatbot more empathetic, while the patient panel preferred physicians [28]. The increase in empathy scores when the response was thought to have been written by a physician [29] and cancer survivors reporting a lack of human empathy in AI as a fundamental limitation [12] indicate that perceived empathy in the text and empathy experienced in relation are not interchangeable. Therefore, the evaluation of empathy depends not only on the content of the information, but also on who and in what relationship it is presented.

The comprehensibility of medical information also varied according to the context of comparison. Although the readability level of chatbot responses was found to be low in objective analyses [10,30,31], participants evaluated AI as a tool that simplifies medical knowledge. This discrepancy may arise from comparing responses to hard-to-understand pathology reports, rather than general literacy metrics. However, the fact that information is understandable does not mean that it is accurate, reliable, or personal; moreover, there is a risk of carrying non-personalized information into clinical decision processes [9].

The fact that information provided by AI chatbots was disconnected from the clinical context and personal health history led to a conditional nature of trust in artificial intelligence. It was reported in the literature that patients approached artificial intelligence cautiously, and that trust in these tools was shaped by trust in the physician [5,32,33,34]. It was also suggested that artificial intelligence could mediate mutual trust between patient and physician [35]. Our findings suggest that trust is built through repeated validation processes: overlapping responses with physician descriptions increased trust, while conflicting responses diminished trust and prevented chatbots from being seen as the ultimate source of information. The portrayal of artificial intelligence as a counselor or teacher on the one hand, and as a hard and unreliable tool on the other, reflected this limited and conditional role.

The fact that participants verified information obtained from artificial intelligence against physicians’ explanations has actually highlighted the clinical and relational importance of healthcare professionals, which is contrary to concerns that this technology might weaken the patient–physician relationship [11,36]. This interpretation reflects the comparative behavior and perceptions reported by our participants and should not be read as evidence of a measured causal effect of AI chatbot use on the patient–physician relationship more broadly; confirmation would require controlled or longitudinal designs. On the other hand, a remarkable “paradox of non-sharing” has emerged. Although 77.5% of Turkish medical oncologists reportedly utilized large language models in their professional practice [37], some participants masked their own use out of concern for being viewed negatively or considered disrespectful to the physician. This can make the source of the information obtained from artificial intelligence invisible, making it difficult for the clinician to recognize and correct incorrect or out-of-context content. Similar concerns were reported in a study conducted with oncologists in China [38], suggesting that this pattern may not be unique to Türkiye.

Data privacy, which was reported as a significant concern in surveys conducted in Türkiye [39], did not emerge as an independent theme in this study. However, this should not be interpreted as participants considering privacy unimportant, as privacy was reported as one of the main barriers in cancer survivors [12]. The fact that privacy did not become evident in the interviews indicated that the risks associated with sharing health data with artificial intelligence tools should be clearly addressed in clinical information.

### 4.1. Reflections on Practice

The findings have four main reflections on oncology services and clinical communication. These reflections are exploratory and hypothesis-generating rather than validated clinical guidelines: they are derived from a small, single-center qualitative sample and should be tested in larger, more diverse settings before being adopted as standard practice.

1. **Management of the waiting process:** The time between the delivery of the test result to the patient and its announcement by the clinician should be shortened as much as possible. In cases where this is not possible, short referral notes can be added to patient information systems indicating that the results alone are not sufficient to make a diagnosis or treatment decision.
2. **Non-judgmental communication:** If patients hide the use of artificial intelligence, it may lead to false information being carried into the clinical interview with an unclear source. Asking about this usage in routine and non-judgmental language by healthcare professionals can make it easier to recognize and correct misinformation.
3. **Supporting digital health literacy:** Patients should be supported in composing the right questions to submit to AI chatbots, comparing sources, and verifying information with the clinician. Chatbots should be positioned as supplementary resources that facilitate clinical interview preparation, not as clinician replacement tools.
4. **Incorporating AI use into clinical encounters and addressing misinformation**: Beyond simply asking whether patients have consulted a chatbot, clinicians might invite patients to bring the specific AI-generated content they found confusing or concerning into the consultation so that accurate elements can be affirmed and perceived misinformation addressed together. A single, structured intake question about chatbot use could be integrated into existing pre-visit workflows with little additional time burden. Institutions may also consider providing patients with a short list of vetted, cancer-specific information resources as an alternative to general-purpose chatbots for disease-specific content.

### 4.2. Strengths and Limitations of the Research

The main strength of this study is that it examines the use of chatbots, which is spontaneously initiated and directly related to the disease in the first six months after diagnosis, through real experiences. Independent coding, the classification of selected statements by an independent expert, and participant confirmation support the credibility of the findings.

The fact that this study was conducted in a single center, the sample consisted only of chatbot users, and more than half of the participants had a university-level, or higher, education may limit the transferability of the findings. By design, the inclusion criteria required prior use of an artificial intelligence chatbot; consequently, patients who held negative attitudes toward AI, who lacked access to digital devices or the internet, or whose digital health literacy was too limited to attempt such use were systematically excluded from the sample, and the findings should therefore be interpreted as reflecting the experience of chatbot users specifically rather than cancer patients in general. Furthermore, the sample was dominated by patients with breast cancer (60%) and early-stage disease (stages I–II, 70%), and more than half held a university degree or higher. These characteristics may have shaped the findings in several ways. First, breast cancer is among the most publicly visible cancer types in Türkiye, with established screening and awareness programs; participants with breast cancer may therefore have been more accustomed to actively seeking health information than patients with less publicized cancer types. Second, most participants had early-stage disease, which may have concentrated their concerns on diagnostic uncertainty and prognosis rather than on the more acute symptom burden and existential concerns typical of advanced disease. Third, the relatively high educational level may have helped participants formulate effective questions and critically compare chatbot responses with their physicians’ explanations, which is a strategy that may be less accessible to patients with lower digital or health literacy. Taken together, the findings of this study should be understood as describing the experience of a relatively information-engaged subgroup of chatbot-using cancer patients. In keeping with the descriptive phenomenological design, the findings are offered for readers to judge their transferability rather than as generalizable claims, and should not be extended to patients with advanced disease, less common cancer types, or lower educational attainment without further study.

The fact that the interviews were kept short and that the interviewer had a clinical care relationship with some of the participants may also have influenced the narratives. Emphasizing voluntariness and having the analysis conducted by researchers who are not involved in clinical care are measures to mitigate this impact. In addition, this study did not systematically collect technical parameters of chatbot use, such as the model version, subscription status, the exact prompts submitted, or the content of uploaded medical documents. Because these variables can materially affect chatbot output and the resulting patient experience, their absence limits the interpretive precision of the findings. The external expert check on codebook clarity was likewise based on a small, purposively selected set of example quotations rather than an independent coding of a larger, randomly selected sample of the full transcripts; this narrower scope should be considered when interpreting the reported agreement.

### 4.3. Future Research

Future studies should be carried out with multicenter samples from different education levels, including individuals with low digital health literacy and advanced disease; this will allow for the examination of possible differences in experience between subgroups. Future studies would also benefit from systematically recording the technical parameters of chatbot use, including the model version, subscription tier, the exact prompts submitted, and the type of medical documents uploaded, since these variables are likely to shape the content of AI responses and patients’ subjective experiences related to them. Longitudinal research designs are valuable for understanding how the use of and trust in artificial intelligence evolve over time, spanning the period from initial diagnosis through treatment and survival. Complementary research that examines clinicians’ perspectives on this use can reveal how the pattern of not sharing is experienced from both sides. Finally, there is a requirement for intervention studies examining the impact of the use of non-judgmental language by healthcare professionals and the provision of structured digital health literacy support on patients’ level of openness and clinical outcomes.

## 5. Conclusions

This study showed that artificial intelligence chatbots are a complementary intermediate resource used by individuals diagnosed with cancer to make sense of medical terms, temporarily reduce the uncertainty between the examination result and clinical explanation, and prepare for the physician interview. However, these tools are not alternatives to the therapeutic relationship established with a healthcare professional, as they fail to comprehensively evaluate the patient’s clinical history and examination findings, do not bear professional responsibility, and cannot replace human empathy. The participants did not view artificial intelligence as an ultimate authority; instead, they developed a conditional trust by comparing AI responses with explanations provided by physicians.

The safe and patient-centered use of artificial intelligence in oncology care requires making the digital information-seeking behaviors of patients visible in a non-judgmental communication environment and evaluating the information obtained under the guidance of the clinician.

These conclusions are drawn from a qualitative, single-center sample of patients who had already used AI chatbots. The results of this study should be interpreted as hypothesis-generating insights into a specific patient experience rather than generalizable claims about the effects of AI chatbot use on cancer patients’ anxiety, trust, or patient–physician relationships more broadly. Finally, confirmation in larger and more diverse samples, including experimental or longitudinal designs, is required before these implications inform clinical policy.

## Figures and Tables

**Table 1:** Sociodemographic and clinical characteristics of the participants.

Feature	Female (*n* = 12) *n* (%)	Male (*n* = 8) *n* (%)	Total (*n* = 20) *n* (%)
Age group (years)			
18–34	1 (8)	0 (0)	1 (5)
35–49	8 (67)	1 (13)	9 (45)
50–64	2 (17)	5 (63)	7 (35)
65–79	1 (8)	2 (25)	3 (15)
Education level			
Primary school	2 (17)	1 (13)	3 (15)
Secondary school	1 (8)	1 (13)	2 (10)
High school	3 (25)	1 (13)	4 (20)
University and above	6 (50)	5 (63)	11 (55)
Marital status			
Married	9 (75)	7 (88)	16 (80)
Single	3 (25)	1 (13)	4 (20)
Employment status			
Working	3 (25)	0 (0)	3 (15)
Not working	7 (58)	4 (50)	11 (55)
Retired	2 (17)	4 (50)	6 (30)
Income–expense perception			
Income less than expenses	2 (17)	1 (13)	3 (15)
Income equal to expenses	7 (58)	1 (13)	8 (40)
Income more than expenses	3 (25)	6 (75)	9 (45)
Type of cancer			
Breast	11 (92)	1 (13)	12 (60)
Gastric	0 (0)	2 (25)	2 (10)
Other *	1 (8)	5 (63)	6 (30)
Stage			
I	4 (33)	3 (38)	7 (35)
II	6 (50)	1 (13)	7 (35)
III	1 (8)	3 (38)	4 (20)
IV	1 (8)	1 (13)	2 (10)
Time since diagnosis (months)			
0–2	8 (67)	3 (38)	11 (55)
3–4	3 (25)	2 (25)	5 (25)
5–6	1 (8)	3 (38)	4 (20)
Treatment received **			
Chemotherapy	8 (67)	6 (75)	14 (70)
Surgery	7 (58)	5 (63)	12 (60)
Radiotherapy	5 (42)	0 (0)	5 (25)
Hormone therapy	2 (17)	0 (0)	2 (10)

* Lung, colon, rectum, and ovarian cancers; sarcoma; and pancreatic neuroendocrine tumor (*n* = 1 each). ** Since the participants received more than one treatment, the percentages were calculated based on the sum of the relevant column.

**Table 2:** Digital technology and chatbot use by the study participants.

Feature	Total (*n* = 20) *n* (%)
Use of digital devices	
Daily	18 (90)
Rarely	2 (10)
Chatbot used *	
ChatGPT	14 (70)
Gemini	7 (35)
Claude	1 (5)
Meta AI	1 (5)
When the use started	
Before diagnosis	14 (70)
After diagnosis	6 (30)
Frequency of use	
Only at certain moments	14 (70)
Daily	5 (25)
Weekly	1 (5)
Change in use after diagnosis	
Increased	12 (60)
Unchanged	7 (35)
Decreased	1 (5)

* Three participants reported using more than one tool.

## Data Availability

The qualitative data generated and analyzed during the current study are not publicly available because they contain potentially identifiable and sensitive participant information. De-identified data may be available from the corresponding author upon reasonable request, subject to ethical and institutional requirements.

## Supplementary Materials

The following supporting information can be downloaded at: https://www.mdpi.com/article/10.3390/curroncol33090563/s1, Table S1: Participant-level theme and sub-theme visibility matrix (n = 20).

## Abbreviations

The following abbreviations are used in this manuscript:

AI	Artificial intelligence
COREQ	Consolidated Criteria for Reporting Qualitative Research
LLM	Large language model
MAXQDA	MAXQDA Qualitative Data Analysis Software
