# Supplementary material for: The Use of Artificial Intelligence Chatbots by Newly Diagnosed Cancer Patients: A Descriptive Phenomenological Study
Source: Curr Oncol. 2026 Sep 17;33(9):563. doi: 10.3390/curroncol33090563 (PMC13605247; doi:10.3390/curroncol33090563)
Supplement: Supplementary file 1 [file curroncol-33-00563-s001.zip › curroncol-4534270-supplementary.pdf]

Supplementary Material

Table S1. Participant-level theme and sub-theme visibility matrix (N = 20)

Participant	T1	AT1	AT2	T2	AT3	AT4	T3	AT5	AT6	AT7	T4	AT8	AT9	AT10
K01	1	1	1	1	1	1	1	1	0	1	1	1	0	1
K02	1	1	1	1	1	1	1	1	0	1	1	1	1	1
K03	1	1	1	1	1	1	1	1	1	1	1	1	1	1
K04	1	1	0	1	1	1	1	1	0	1	1	1	1	1
K05	1	1	1	1	1	1	1	1	0	1	1	1	1	1
K06	1	1	1	1	1	1	1	1	0	1	1	1	1	1
K07	1	0	1	1	1	1	1	1	1	1	1	1	1	1
K08	1	1	0	1	1	1	1	1	0	1	1	0	1	1
K09	1	1	1	1	1	1	1	1	1	1	1	1	1	1
K10	1	1	0	1	1	1	1	1	0	1	1	0	1	1
K11	1	1	1	1	1	1	1	1	0	1	1	1	1	1
K12	1	1	1	1	1	1	1	1	0	1	1	1	1	1
K13	1	1	1	1	1	1	1	1	1	1	1	1	1	1
K14	1	0	1	1	1	1	1	1	0	1	1	1	1	1
K15	1	1	1	1	1	1	1	1	0	1	1	1	1	1
K16	1	1	1	1	1	1	1	1	1	1	1	1	1	1
K17	1	1	1	1	1	1	1	1	1	1	1	1	1	1
K18	1	0	1	1	1	1	1	1	0	1	1	1	1	1
K19	1	1	1	1	1	1	1	1	1	1	1	1	1	1
K20	1	1	1	1	1	1	1	1	1	1	1	1	1	1
n (%)	20(100)	17(85)	17(85)	20(100)	20(100)	20(100)	20(100)	20(100)	8(40)	20(100)	20(100)	18(90)	19(95)	20(100)

Note: 1 indicates that the participant has at least one coded expression under the relevant theme or subtheme; 0 indicates that it does not

Theme and child theme key.

T1: Purposes of using artificial intelligence; **AT1:** Artificial intelligence as the first source of information acquisition; **AT2:** Treatment process and management of side effects.**T2:** Experience of interacting with artificial intelligence; **AT3:** Emotional effects of the use of artificial intelligence; **AT4:** Trust in AI interaction. **T3:** Reflection of artificial intelligence experience on the relationship with the treatment team; **AT5:** The vitalness of the health professional; **AT6:** Attending the interview prepared; **AT7:** The dynamic of sharing the usage experience. **T4:** Evaluation of the use of artificial intelligence; **AT8:** Perceived benefits; **AT9:** Perceived limitations of AI; **AT10:** Recommendations for use.
